# Organ-Specific Chemical Diversity and Biofunctional Potential of *Ebenus laguroides* subsp. *laguroides*: Linking Phenolic Composition with Antioxidant and Enzyme Inhibitory Activities

**DOI:** 10.3390/molecules31050826

**Published:** 2026-02-28

**Authors:** Bedrettin Selvi

**Affiliations:** Department of Biology, Faculty of Arts and Sciences, Tokat Gaziosmanpaşa University, 60250 Tokat, Türkiye; bedrettin.selvi@gop.edu.tr; Tel.: +90-5306923749

**Keywords:** *Ebenus laguroides* subsp. *laguroides*, phenolic profile, LC-ESI-MS/MS, antioxidant activity, enzyme inhibition, RACI, correlation analysis

## Abstract

Plants adapted to gypsum-rich habitats often display unique metabolic specializations. This study investigated the organ-specific chemical diversity and biofunctional potential of *Ebenus laguroides* subsp. *laguroides*, a gypsum-endemic legume from Central Anatolia. Methanolic extracts of flowers, leaves, stems, and roots were analyzed for phenolic composition by LC–ESI–MS/MS and evaluated for antioxidant and enzyme inhibitory activities. Twenty-one phenolics were identified, dominated by hesperidin, verbascoside, and (+)-catechin, particularly abundant in stems. Stems exhibited the highest total phenolic (82.60 mg GAEs/g) and flavonoid (45.79 mg QEs/g) contents, correlating strongly with antioxidant capacity across multiple assays (r > 0.95). Enzyme inhibition tests revealed moderate but consistent activities, with roots showing the strongest acetylcholinesterase inhibition and stems the highest tyrosinase inhibition. Correlation analyses confirmed strong links between phenolic content, antioxidant potential, and enzyme modulation. The results highlight distinct organ-dependent metabolite patterns and demonstrate that *E. laguroides* subsp. *laguroides* is a noteworthy source of multifunctional phenolics. These findings contribute to understanding the chemical biodiversity and bioactivity relationships within Fabaceae species adapted to gypsum soils and provide a foundation for further phytochemical and pharmacological exploration.

## 1. Introduction

Natural products derived from plants are one of the most important sources of biologically active molecules that are crucial for human health. Among plant-derived bioactive compounds, phenolic compounds have attracted considerable attention due to their widespread occurrence and diverse biological activities [1]. Their role in reducing oxidative stress, in particular, has increased interest in species rich in phenolic compounds. This is even more evident in the Fabaceae family, which boasts a high chemical diversity and is frequently used in folk medicine.

The chemical structure of phenolic compounds directly determines their biological effects. The hydroxyl groups attached to their aromatic rings give these compounds the ability to neutralize free radicals, bind metal ions, and inhibit oxidative chain reactions. Consequently, phenolics may exhibit potent antioxidant, anti-inflammatory, antimicrobial, and cytoprotective properties [1,2,3]. For example, quercetin, a common flavonoid, can reduce cytokine production by regulating inflammation-related signals in both cell cultures and living systems. This suggests that phenolic compounds may have an important biological role in combating chronic diseases [3,4].

The genus *Ebenus*, a member of the Fabaceae family, is known for its species distributed particularly in the Eastern Mediterranean and Western Asian regions. In the Turkish flora, it is abundant in the Mediterranean and Central Anatolian zones [5]. Recent studies have revealed that *Ebenus* species possess rich phenolic contents and exhibit antioxidant and antimicrobial effects associated with these compounds. For example, *E. hirsuta* extracts have been reported to exhibit significant antimicrobial and enzyme inhibition activities, consistent with phenolic compound profiles determined by LC–MS/MS analyses [6]. Similarly, studies on *E. pinnata* have shown that the plant possesses strong antioxidant and antibacterial properties [7].

*Ebenus laguroides* Boiss. is a local endemic species that spreads in the Central Anatolian region of Turkey, especially in the gypsum soils around Sivas. *E. laguroides* subsp. *laguroides* is one of the rare plants that grows on gypsum slopes and is considered among the gypsum endemics in the Sivas flora [8]. Gypsum soils, thanks to their chemically unique structures, facilitate the development of different metabolites in plants. However, there is very little information about the chemical composition and biological effects of this plant. In some studies at the species level, the phenolic contents of *E. laguroides* have been determined and its antioxidant and antimicrobial activities have been reported [9]. However, a comprehensive phytochemical or biological activity study at the subspecies level has not yet been encountered.

This lack of information is important for two reasons. First, the amount and composition of phenolic compounds can vary significantly from plant to plant, even between different organs of the same plant. These differences are closely linked to environmental factors such as habitat conditions, microclimate, soil chemistry (e.g., gypsum-rich environments), and developmental stage [10,11]. Second, plants adapted to gypsum-rich areas often have specific metabolic profiles specific to sulfur- and calcium-rich soils [12]. Therefore, examining different plant parts separately is crucial for understanding chemical diversity.

This study aims to evaluate methanolic extracts obtained from the leaves, stems, flowers, and roots of *E. laguroides* subsp. *laguroides* in detail for the first time in terms of phenolic composition, antioxidant potential, and enzyme inhibitory activities. Total phenolic and flavonoid contents, LC–ESI–MS/MS-based phenolic profiling, antioxidant assays, and enzyme inhibition assays were performed to comprehensively evaluate the phytochemical composition and biofunctional properties of this endemic subspecies. The primary objectives of the study were to expand the current knowledge of the phenolic constituents of *E. laguroides* subsp. *laguroides*, to elucidate the relationship between organ-specific phenolic diversity and biofunctional activity, and to position this subspecies within the limited phytochemical framework of the *Ebenus* genus. The findings are expected to contribute to the phytochemical characterization of Fabaceae species growing in gypsum-rich environments and to support future pharmacological and conservation-oriented research on geographically restricted Anatolian taxa.

It is important to emphasize that although *E. laguroides* subsp. *laguroides* is naturally restricted to gypsum-rich habitats, the present study does not experimentally evaluate gypsum-driven metabolic adaptation. Gypsum soils are characterized by high calcium sulfate content, low water retention capacity, and nutrient imbalance, which may influence plant secondary metabolism through abiotic stress signaling pathways [13,14]. However, without comparative analyses involving non-gypsum populations or closely related taxa from contrasting edaphic environments, causal inferences regarding gypsum-induced metabolic specialization cannot be established. Therefore, the gypsum habitat is considered here primarily as ecological context rather than as a testable mechanistic driver of metabolite biosynthesis. The primary focus of this research is the comparative phytochemical characterization and biofunctional evaluation of phenolic compounds across different plant organs under natural conditions. The antioxidant and enzyme inhibitory assays employed in this study represent widely accepted functional screening tools for assessing bioactive potential but do not directly reflect specific physiological mechanisms of gypsum stress adaptation. Future comparative and mechanistic investigations focusing on osmolytes, sulfur-containing metabolites, calcium-associated signaling pathways, and populations from contrasting soil environments would be required to elucidate the physiological and ecological drivers of gypsum adaptation [10,11,15].

## 2. Results and Discussion

### 2.1. Chemical Composition

**Figure 1:**
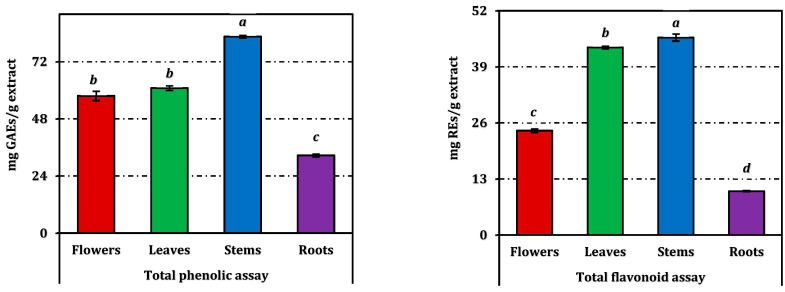
Total phenolic and flavonoid contents of *E. laguroides* subsp. *laguroides* extracts. GAEs and REs: Gallic acid and rutin equivalents, respectively. Values indicated by the same superscripts (a–d) within the same column are not significantly different according to Tukey’s HSD test at the 5% significance level.

Total phenolics (TPC) and flavonoids (TFC) varied markedly among organs (Figure 1). Stems yielded the highest mean TPC (82.60 ± 0.51 mg GAEs/g), followed by leaves (60.97 ± 0.98 mg GAEs/g) and flowers (57.66 ± 2.01 mg GAEs/g), yet these three extracts did not differ statistically; roots displayed a significantly lower TPC (32.65 ± 0.61 mg GAEs/g). In contrast, TFC showed a clear gradient (all pairwise different): stems > leaves > flowers > roots, with values of 45.79 ± 0.84, 43.51 ± 0.30, 24.21 ± 0.43, and 10.16 ± 0.11 mg REs/g extract, respectively (Figure 1).

Targeted LC-ESI-MS/MS profiling resolved 28 phenolics, 21 of which were detected (Table 1). Hesperidin dominated across all organs and peaked in stems (18,016 µg/g), exceeding leaves (13,969 µg/g), flowers (11,819 µg/g), and roots (3788 ± 37 µg/g). Stems were further distinguished by elevated flavan-3-ols—(+)-catechin (4.434 µg/g) and (−)-epicatechin (2238 µg/g)—and by the highest verbascoside (582 µg/g), indicating a stem-biased accumulation of flavonoid and phenylethanoid glycosides.

Flowers were comparatively enriched in several benzoic/cinnamic acid derivatives and aglycones, including protocatechuic (429 µg/g), *p*-coumaric (108 µg/g), 3-/4-hydroxybenzoic (100–101 µg/g), ferulic (81.5 µg/g), syringic (57.8 µg/g), gallic (49.9 µg/g), quercetin (48.2 µg/g), rosmarinic (26.8 µg/g), and caffeic acid (19.9 µg/g).

Roots exhibited a distinct phenolic signature characterized by maxima in vanillin (61.0 µg/g), chlorogenic acid (103 µg/g), luteolin-7-glucoside (15.7 µg/g), sinapic acid (13.1 µg/g), and eriodictyol (3.69 µg/g), alongside second-highest levels for several hydroxybenzoates. Leaves generally showed lower concentrations for most targets, with notable exceptions such as hesperidin and rosmarinic acid (17.8 µg/g) (second-highest).

These data indicate stem-driven flavonoid richness consistent with the highest TFC, flower-biased abundance of multiple phenolic acids and aglycones despite comparable TPC to stems/leaves, and a root-specific profile dominated by chlorogenic/vanillin-type phenolics and select flavonoid glycosides (Figure 1; Table 1).

The phytochemical profile of *E. laguroides* subsp. *laguroides* revealed a marked organ-specific differentiation, highlighting the biochemical specialization of the species. The predominance of flavonoid-type compounds in the stems, particularly hesperidin, catechin, epicatechin, and verbascoside, reflects a metabolic allocation pattern consistent with protective and signaling functions in aerial tissues. Similar flavonoid enrichment in aboveground organs has been documented in other *Ebenus* taxa, including *E. pinnata* and *E. haussknechtii*, where compounds such as rutin, kaempferol-3-O-rutinoside, quercetin glycosides, and catechin were isolated from aerial parts [16,17]. These compounds are typically associated with defense against oxidative stress, ultraviolet radiation, and herbivory—factors that are particularly relevant to Mediterranean and semi-arid ecosystems where *Ebenus* species thrive.

The compositional similarity between stems and leaves in total phenolics but not in individual compound patterns suggests tissue-specific fluxes through the phenylpropanoid pathway. Bektaş et al. [9] reported comparable phenolic constituents—gallic, protocatechuic, *p*-hydroxybenzoic, vanillic, and *p*-coumaric acids—in the leaves and flowers of *E. laguroides*, supporting the general consistency of phenolic acid profiles within the species. However, in the present subspecies, several phenolic acids such as ferulic, syringic, and rosmarinic acids were also detected at notable levels, implying subtle biochemical divergences likely attributable to organ differentiation and environmental adaptation to gypsum-rich habitats. Such ecological specialization can influence secondary metabolism through altered redox homeostasis and nutrient availability, as reported for gypsum-adapted legumes.

The dominance of hesperidin across all organs, with maximal accumulation in stems, mirrors the pattern reported for *E. hirsuta*, which contained high hesperidin and rutin levels [6]. This parallel suggests a genus-level metabolic trend favoring flavanone glycosides as key antioxidant and regulatory metabolites. Moreover, the co-occurrence of catechin and epicatechin in stems of *E. laguroides* subsp. *laguroides* aligns with findings in *E. pinnata*, where catechin was isolated as a major antioxidant compound [17]. These observations collectively highlight the conserved role of flavan-3-ols within *Ebenus* species as radical scavengers contributing to cellular protection.

Flowers of the studied subspecies exhibited a rich array of hydroxybenzoic and hydroxycinnamic acids, including protocatechuic, *p*-coumaric, and ferulic acids—compounds also dominant in the aerial parts analyzed by Bektaş et al. [9]. Such phenolic acids are frequently implicated in pollination-related pigmentation and antimicrobial defense, consistent with the ecological roles of floral tissues. The root extracts, on the other hand, presented a distinct profile with elevated chlorogenic and vanillin-type phenolics, together with luteolin-7-glucoside. Root-enriched chlorogenic acid has been associated with allelopathic and defense mechanisms in soil-contacting tissues, suggesting adaptive biochemical diversification.

Taken together, the chemical fingerprint of *E. laguroides* subsp. *laguroides* integrates the key metabolic trends previously reported for other *Ebenus* species—namely the predominance of flavonoid glycosides, phenolic acids, and select flavan-3-ols—yet exhibits a more organ-differentiated accumulation pattern. The prevalence of verbascoside in stems further extends the known metabolite spectrum of the genus, since this compound had not been highlighted in earlier studies of *Ebenus* [6,9]. Collectively, these findings point to a structurally diverse phenolic system shaped by both phylogenetic inheritance and environmental constraints. Quantitatively, the phenolic concentrations observed in *E. laguroides* subsp. *laguroides* are comparable to or exceed those reported in related *Ebenus* species and other Fabaceae taxa. For instance, Bektaş et al. [9] reported total phenolic contents ranging from 45.2 to 67.8 mg GAE/g extract in *E. laguroides* aerial parts, which are lower than the stem phenolic content observed in the present study (82.60 mg GAE/g). Similarly, Ceylan et al. [6] reported total phenolic contents of approximately 52–74 mg GAE/g in *E. hirsuta* extracts, again slightly lower than the maximum values observed here. In addition, studies on *E. pinnata* demonstrated total phenolic levels between 40 and 70 mg GAE/g depending on plant organ and ecological conditions [7]. These comparisons indicate that *E. laguroides* subsp. *laguroides*, particularly its stem tissues, represents a relatively rich source of phenolic compounds within the genus. Such quantitative differences may reflect species-specific metabolic regulation, organ specialization, and environmental influences, further highlighting the phytochemical distinctiveness and biological relevance of this endemic subspecies. In addition to total phenolics, the exceptionally high hesperidin (18,016 µg/g) and catechin (4434 µg/g) concentrations detected in stems further emphasize the metabolic intensity of this organ compared with previously reported *Ebenus* taxa. The notable accumulation of verbascoside (582 µg/g) also expands the documented metabolite spectrum of the genus. These quantitative distinctions reinforce the pronounced organ-driven specialization observed in the present subspecies.

### 2.2. Antioxidant Activity

**Figure 2:**
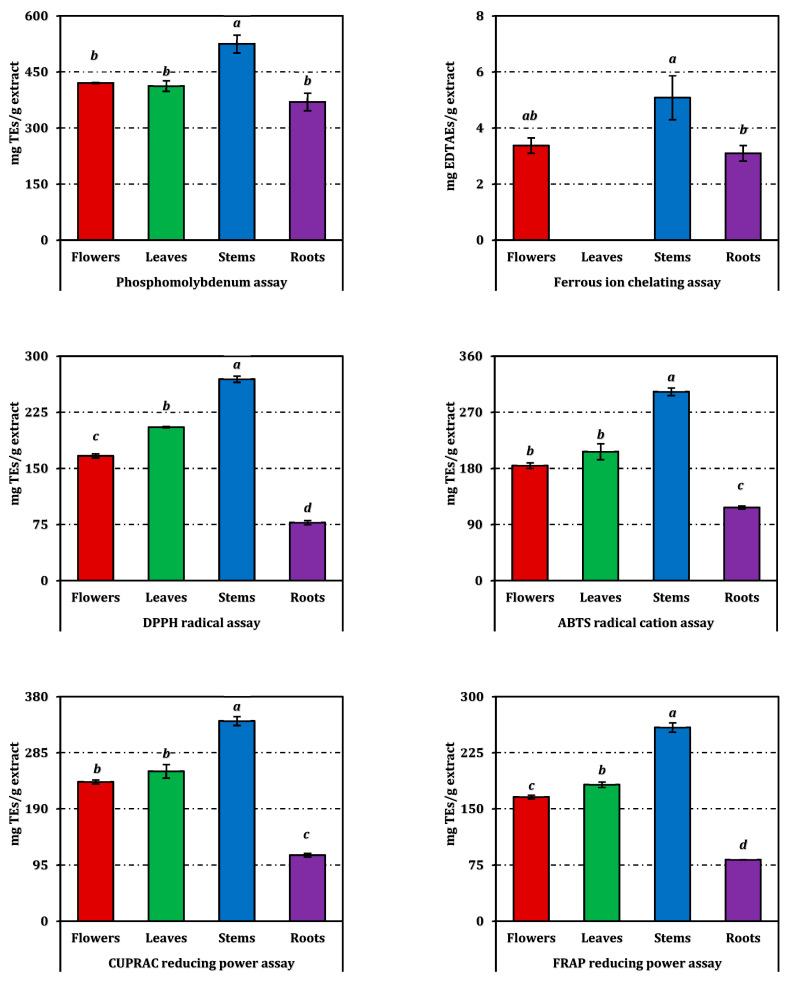
Antioxidant activity of *E. laguroides* subsp. *laguroides* extracts. TEs and EDTAEs, trolox and ethylenediaminetetraacetic acid (disodium salt) equivalents, respectively. Values indicated by the same superscripts (a–d) on the bar chart are not significantly different according to Tukey’s HSD test at the 5% significance level.

The antioxidant potential of the *E. laguroides* subsp. *laguroides* extracts was evaluated through six complementary in vitro assays, and the results are summarized in Table 2. Additional comparative data expressed in positive control equivalents are presented in Figure 2 for reference. Among the tested samples, the stem extract consistently exhibited the strongest antioxidant performance across almost all assays, followed by the leaf and flower extracts, whereas the root extract demonstrated the weakest activity overall.

In the phosphomolybdenum assay, the stem extract (EC_50_: 0.85 mg/mL) showed the highest total antioxidant capacity, significantly surpassing the other parts (*p* < 0.05). The flower and leaf extracts displayed comparable activity (1.06 mg/mL), while the root extract (1.19 mg/mL) was the least potent. A similar pattern was observed in the reducing power assays (CUPRAC and FRAP), where stems again produced the lowest EC_50_ values (0.51 and 0.19 mg/mL, respectively), indicating superior electron-donating ability. Roots exhibited markedly weaker reducing capacity in both tests (1.55 mg/mL and 0.60 mg/mL, respectively).

Free-radical scavenging activities, assessed by DPPH and ABTS assays, also highlighted the stem extract as the most effective (IC_50_: 0.94 mg/mL for DPPH and 0.58 mg/mL for ABTS), while roots required substantially higher concentrations to reach 50% inhibition (3.29 mg/mL and 1.49 mg/mL, respectively). The leaf and flower extracts displayed moderate radical scavenging capacities, consistent with their intermediate phenolic contents.

**Figure 3:**
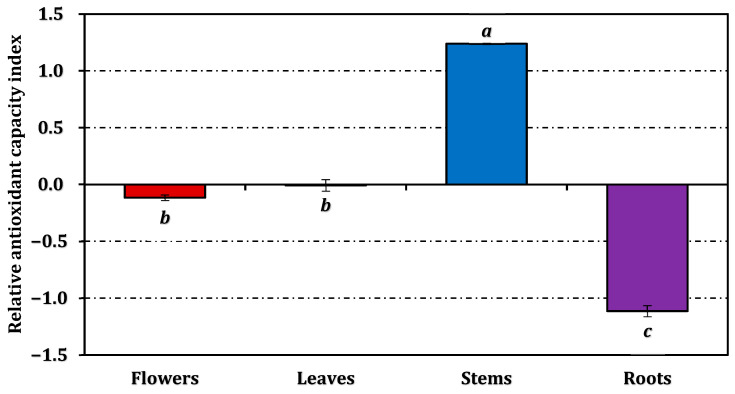
Relative antioxidant capacity index of *E. laguroides* subsp. *laguroides* extracts. Values indicated by the same superscripts (a–c) on the bar chart are not significantly different according to Tukey’s HSD test at the 5% significance level.

**Figure 4:**
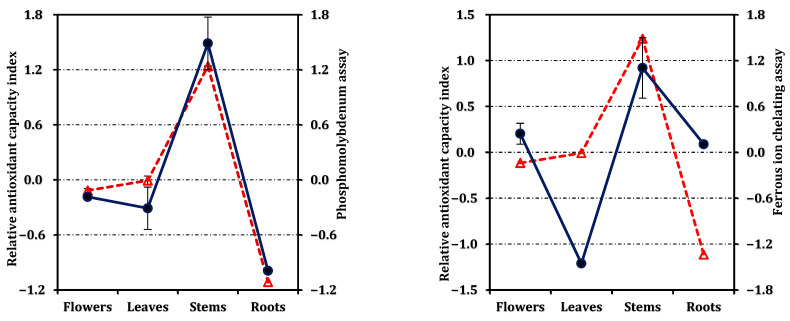

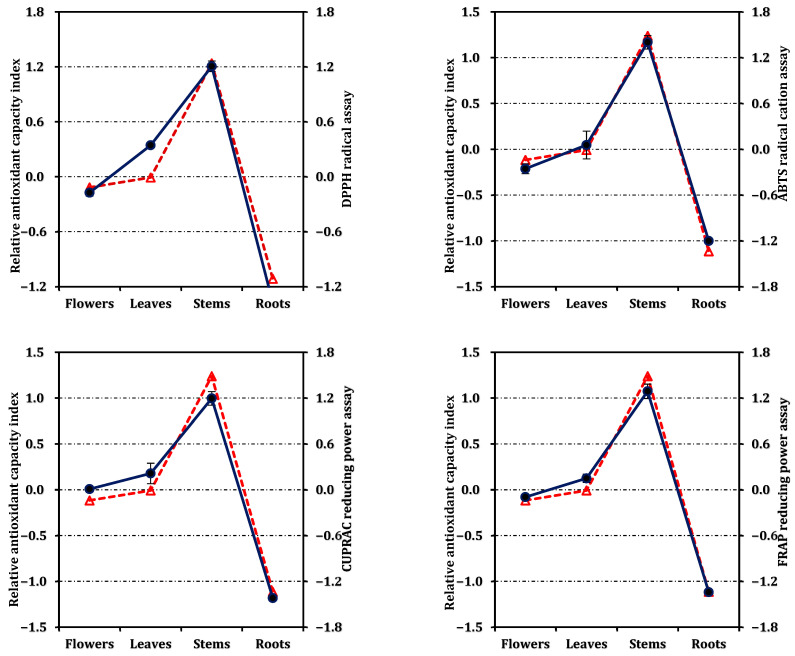
Correlation between the relative antioxidant capacity index (dashed red line with triangle) and antioxidant activity (solid dark blue line with circle).

The ferrous ion chelating assay revealed a distinct trend: the stem extract (IC_50_: 3.84 mg/mL) showed measurable metal chelation, whereas the leaf extract was inactive in this test. Flowers and roots exhibited comparable, though limited, chelating capacity (5.74 mg/mL and 6.26 mg/mL, respectively). The overall antioxidant efficiency, expressed as the RACI, followed the same hierarchy—stems > leaves ≈ flowers > roots (Figure 3). The RACI values were strongly correlated with most antioxidant parameters (Figure 4), except for a negative correlation observed between the ferrous ion chelating capacity of stems and leaves and their corresponding RACI scores.

The antioxidant behavior of *E. laguroides* subsp. *laguroides* extracts revealed a clear organ-dependent trend, with the stem extract consistently outperforming the others across most assays. This pattern strongly parallels the distribution of total phenolic and flavonoid contents observed in the species, underscoring the crucial role of phenolic compounds in the plant’s antioxidant potential. The strong reducing and radical scavenging abilities of the stem extract indicate a high electron- and hydrogen-donating capacity, typical of phenolic-rich matrices. Such a correlation between phenolic abundance and antioxidant strength has been widely reported in other *Ebenus* taxa as well [6,9].

In earlier studies, *E. laguroides* methanolic extracts were also characterized by substantial antioxidant capacity, particularly in DPPH and FRAP assays [9]. The consistency between those findings and the present results suggests that this species, regardless of its geographical origin or subspecific variation, is an effective reservoir of redox-active phytochemicals. Moreover, the predominance of the stem fraction as the most potent organ may be attributed to its structural and metabolic role, as stems often accumulate phenolic polymers and flavonoids associated with mechanical and oxidative stress protection.

Comparable antioxidant patterns have been documented in *E. pinnata* across different ecotypes, where plants from semi-arid regions exhibited markedly higher phenolic concentrations and radical scavenging efficiency than those from humid environments [7]. This geographical influence emphasizes the ecological plasticity of *Ebenus* species and supports the hypothesis that plants adapted to more stressful or xeric conditions tend to reinforce their antioxidant systems as a physiological defense mechanism. The strong antioxidant performance observed in *E. laguroides* subsp. *laguroides*, collected from gypsum-rich habitats, may be consistent with enhanced phenolic reinforcement under ecologically constrained environments, although direct adaptive mechanisms were not experimentally evaluated in this study.

Although gypsum-rich habitats are known to influence plant secondary metabolism due to their unique physicochemical characteristics, the present study does not directly test habitat-driven metabolic differentiation. Therefore, the observed phenolic distribution patterns should be interpreted primarily as organ-specific biochemical variation rather than as direct evidence of gypsum-driven metabolic adaptation. Future comparative studies involving related species or populations from contrasting soil environments would be required to clarify the ecological drivers of these phytochemical patterns [10,11].

The current findings are also consistent with the results obtained for other congeneric species. For instance, methanolic extracts of *E. hirsuta* displayed remarkable activity in DPPH, ABTS, and CUPRAC assays [6], while *E. haussknechtii* flower extracts exhibited potent radical scavenging comparable to standard antioxidants [18]. The comparable assay profiles among these taxa suggest a conserved antioxidative mechanism within the genus, likely mediated by structurally related flavonoids and phenolic acids such as rutin, catechin, and kaempferol glycosides, which were previously isolated from *E. pinnata* [17]. These molecules are well known for their synergistic interactions in neutralizing reactive oxygen species and stabilizing free radicals through electron donation and resonance delocalization mechanisms [19,20].

Interestingly, while most antioxidant parameters followed a coherent trend among organs, the metal chelation capacity did not align with reducing power or radical scavenging indices. This divergence, also reported in other *Ebenus* species [6], likely arises from differences in chelating group density and metal-binding affinity of the phenolic constituents. The relatively weak chelation observed here might thus be compensated by the strong electron transfer ability of the same compounds.

These findings confirm that *E. laguroides* subsp. *laguroides* represents a noteworthy natural antioxidant source within the *Ebenus* genus. The strong redox performance, especially in the stem and leaf extracts, can be ascribed to the high accumulation of multifunctional phenolics capable of acting through diverse antioxidant pathways, including radical scavenging, reducing, and total capacity mechanisms. This multi-assay approach not only substantiates the robustness of its antioxidant profile but also aligns with previous reports highlighting *Ebenus* species as promising candidates for nutraceutical and pharmaceutical applications.

### 2.3. Enzyme Inhibitory Activity

**Figure 5:**
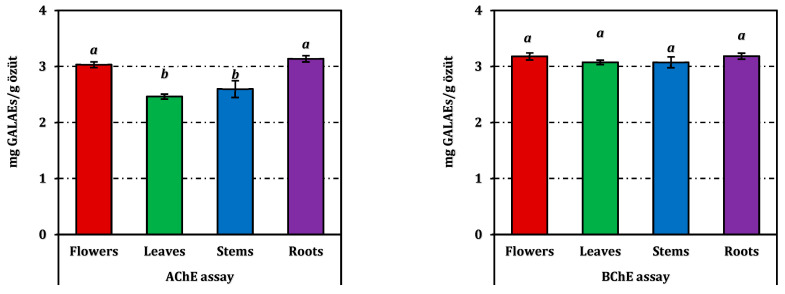

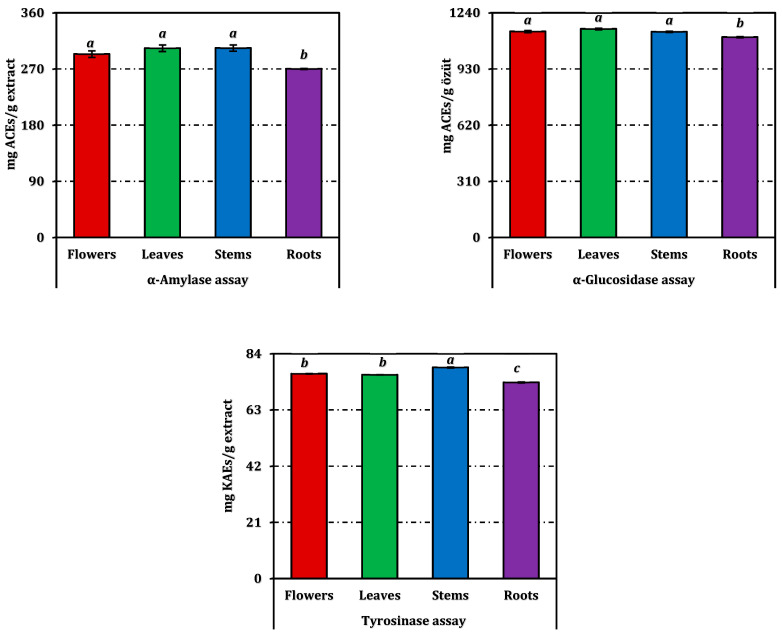
Enzyme inhibition activity of *E. laguroides* subsp. *laguroides* extracts. ACEs, GALAEs and KAEs mean acarbose, galanthamine and kojic acid equivalents, respectively. Values indicated by the same superscripts (a–c) on the bar chart are not significantly different according to Tukey’s HSD test at the 5% significance level.

The enzyme inhibitory potential of *E. laguroides* subsp. *laguroides* extracts is summarized in Table 3, while a comparative visualization in terms of positive control equivalents is provided in Figure 5. All extracts demonstrated a moderate level of inhibition across the tested enzyme systems, though the extent varied depending on the enzyme type and plant organ.

Regarding cholinesterase inhibition, both AChE and BChE assays revealed comparable patterns among the extracts. However, the inhibitory activity of the extracts against BChE was found to be slightly stronger than that against AChE. The root extract exhibited the strongest AChE inhibition (IC_50_ = 1.02 mg/mL), closely followed by the flower extract (1.06 mg/mL). In contrast, the leaves and stems showed slightly weaker inhibition, with IC_50_ values of 1.30 and 1.23 mg/mL, respectively. Although all extracts were markedly less potent than the reference inhibitor galanthamine (IC_50_ = 0.0032 mg/mL for AChE and 0.0031 mg/mL for BChE), these results still indicate meaningful anticholinesterase potential.

For tyrosinase inhibition, IC_50_ values ranged narrowly from 1.05 mg/mL (stems) to 1.13 mg/mL (roots), suggesting a consistent but moderate activity across organs. However, all plant extracts were substantially less active than kojic acid (IC_50_ = 0.082 mg/mL).

Concerning carbohydrate-hydrolyzing enzymes, all extracts inhibited α-amylase with IC_50_ values between 3.10 and 3.48 mg/mL, and α-glucosidase with values around 1.00 mg/mL. Although weaker than acarbose (IC_50_ = 0.95 mg/mL for α-amylase and 1.12 mg/mL for α-glucosidase), the observed inhibition levels suggest a potential modulatory effect on carbohydrate metabolism.

While none of the extracts approached the potency of the respective standard inhibitors, the results highlight a broad-spectrum yet balanced enzyme inhibitory profile, particularly notable in the root and flower extracts. Detailed comparisons expressed as positive control equivalents can be examined in Figure 5.

The current findings reveal that *E. laguroides* subsp. *laguroides* exhibits a moderate yet wide-ranging inhibitory profile against all tested enzyme systems, including cholinesterases, tyrosinase, and carbohydrate-hydrolyzing enzymes. Although there are no prior reports describing the enzyme inhibitory potential of this taxon, the results agree, at least partially, with observations on *E. hirsuta*, another species of the genus reported to possess measurable inhibitory effects on AChE, BChE, and carbohydrate-metabolizing enzymes [6]. The comparable response pattern across the two *Ebenus* species suggests that members of this genus may share similar bioactive chemical scaffolds capable of targeting multiple enzyme systems.

Among the examined enzymes, the relatively stronger inhibition observed against BChE than AChE in *E. laguroides* could reflect the differential affinity of specific flavonoids and phenolic acids present in the extracts. LC-ESI-MS/MS analysis demonstrated the abundance of compounds such as hesperidin and catechin, both of which have previously been reported to interact with the catalytic anionic site of cholinesterases and thereby attenuate enzymatic activity [21,22]. The moderate cholinesterase inhibition recorded here thus appears consistent with the known pharmacological profiles of these phenolics, indicating that they might act synergistically within the complex plant matrix.

In relation to tyrosinase inhibition, all extracts displayed consistent but modest activity. Similar inhibitory effects have been attributed to flavonoids containing hydroxylated aromatic rings, which can chelate the copper ions at the active site of tyrosinase [23,24]. The presence of hesperidin in the analyzed extracts likely contributes to this activity, as this compound has been demonstrated to suppress tyrosinase through competitive binding to its catalytic domain [23]. The moderate inhibition detected across organs may thus stem from additive contributions of multiple phenolic components rather than from a single dominant inhibitor.

The inhibition of α-amylase and α-glucosidase observed in this study further underscores the functional diversity of the *E. laguroides* extracts. The detected catechins, in particular, are well documented for their capacity to retard carbohydrate digestion by binding to the active sites of these enzymes and reducing substrate accessibility [25,26]. Such inhibition patterns, though weaker than those of acarbose, may nevertheless contribute to postprandial glucose regulation, suggesting a potential nutraceutical relevance of this species.

The enzyme inhibitory actions of *E. laguroides* subsp. *laguroides* likely arise from the combined effects of its abundant flavonoids and phenolic acids, notably catechin, hesperidin, and related derivatives. These constituents appear to act via multiple mechanisms—including metal chelation, active-site occupation, and hydrogen-bond formation—resulting in a balanced multi-target inhibition. Considering the absence of previous data on this subspecies, the present results represent the first evidence of its enzyme-modulating capacity and highlight its potential as a natural source of mild but broad-spectrum inhibitors of enzymes associated with neurodegenerative, pigmentation, and metabolic disorders.

### 2.4. Correlations Among Phenolic Compounds and Assays

The correlation matrix (Table 4) revealed strong and consistent relationships among most antioxidant assays, indicating that these tests generally measured comparable aspects of redox behavior. Notably, FRAP showed the closest association with DPPH (*r* = 0.992), ABTS (*r* = 0.986), and CUPRAC (*r* = 0.990), underscoring the coherence between single electron transfer-based mechanisms. Similarly, RACI, a composite indicator of antioxidant capacity, exhibited excellent correlation with ABTS (*r* = 0.995), FRAP (*r* = 0.993), and total antioxidant power (*r* = 0.956), confirming that the phenolic richness of the extracts was the primary determinant of their antioxidant performance. In contrast, the ferrous ion chelating assay correlated only weakly with the other methods (*r* < 0.40), reflecting its distinct mechanism of action based on metal complexation rather than electron donation.

Among enzyme inhibition parameters, AChE and BChE inhibitory activities showed moderate to strong negative correlations with both antioxidant assays and phenolic content (*r* = −0.56 to −0.79), suggesting that compounds contributing to antioxidant potential may act through mechanisms distinct from cholinesterase inhibition. Conversely, tyrosinase inhibition displayed strong positive correlations with most antioxidant assays (*r* > 0.92 with DPPH, ABTS, and FRAP), as well as with total phenolic and flavonoid contents, implying that redox-active phenolics were likely involved in this enzyme modulation.

The individual phenolics also exhibited notable trends. Total phenolic and flavonoid contents correlated strongly with nearly all antioxidant indices, particularly with FRAP (*r* = 0.999) and DPPH (*r* = 0.951). Among quantified compounds, hesperidin and (+)-catechin showed the most pronounced positive correlations with antioxidant parameters (*r* = 0.984–0.993 and *r* = 0.825–0.957, respectively), while (–)-epicatechin displayed a unique pattern—highly related to metal chelation (*r* = 0.980) but weakly associated with radical scavenging. These findings indicate that different phenolic constituents contribute variably to distinct antioxidant and enzyme inhibition pathways. Overall, the correlation data emphasize the dominant role of total phenolics and specific flavonoids, particularly hesperidin and catechin, in shaping both the antioxidant and bioactive potential of *E. laguroides* subsp. *laguroides* extracts.

## 3. Materials and Methods

### 3.1. Plant Material

Specimens of *E. laguroides* subsp. *laguroides* were collected at full flowering on 1 August 2025, from gypsum-rich habitats near Kümbet village in the Zara district of Sivas, Turkey (39°47′15″ N, 37°49′08″ E), at an elevation of 1895 m. The species was taxonomically identified by Dr. Bedrettin Selvi, and a voucher specimen (herbarium code GOPU 9623) was deposited in the Herbarium of the Faculty of Arts and Sciences, Tokat Gaziosmanpaşa University. In this study, four anatomical parts of the plant—leaves, stems, flowers, and roots—were investigated. Each part was collected separately during harvest. The plant material was air-dried under shade for several weeks, after which the dried samples were finely pulverized using a laboratory mill and stored for subsequent analyses.

### 3.2. Methanol Extraction

Methanolic extraction was performed using 100% methanol (analytical grade). The dried and powdered plant materials from each organ (flowers, leaves, stems, and roots) were extracted separately using ultrasound-assisted extraction in a sonication bath at 30 °C for 60 min, with solvent-to-sample ratio of 20:1 (mL solvent per g dry plant material, *v*/*w*) [27]. The extraction was performed once for each sample. Following sonication, the extracts were filtered and concentrated under reduced pressure using a rotary evaporator. The resulting crude extracts were stored at 4 °C until further analysis. The extraction yields for methanol extracts obtained from flowers, leaves, stems, and roots were 7.31%, 6.11%, 5.47%, and 3.96%, respectively.

### 3.3. Determination of the Phenolic Composition

Total phenolic and flavonoid contents of the extracts were quantified spectrophotometrically following established methods [28]. For LC–ESI–MS/MS analysis, each extract was dissolved in methanol and diluted to an appropriate concentration to ensure compatibility with the calibration range of the analytical method. The diluted solutions were filtered through a 0.22 μm PTFE syringe filter prior to injection into the LC–MS/MS system. Individual phenolic compounds were identified and quantified using a validated LC–ESI–MS/MS method as previously described [29]. Detailed analytical conditions and representative LC–ESI–MS/MS chromatograms of methanolic extracts obtained from the four different plant organs (flowers, leaves, stems, and roots) are provided in the Supplementary Materials (Tables S1 and S2 and Figure S1).

### 3.4. Biological Activity

Antioxidant activities of the extracts were assessed using a series of well-established assays [28,30,31,32,33]. Enzyme inhibitory activities against acetylcholinesterase, butyrylcholinesterase, α-amylase, α-glucosidase, and tyrosinase were evaluated according to previously reported in the literature, with minor modifications. [34,35]. Full methodological details are provided in the Supplementary File.

### 3.5. Statistical Analysis

All experimental data were expressed as mean ± standard deviation (SD). Differences among groups were evaluated by one-way ANOVA followed by Tukey’s post hoc test, with statistical significance set at *p* < 0.05. Analyses were performed using SPSS software (version 26.0).

To assess relationships between measured parameters, Pearson’s correlation coefficients were calculated. Owing to the differing reaction mechanisms of antioxidant assays, direct comparison of absolute values was not appropriate. Therefore, the Relative Antioxidant Capacity Index (RACI) was computed to normalize and integrate results from various assays. RACI values were calculated by subtracting the mean and dividing by the standard deviation of each assay dataset. Correlations between RACI values and individual antioxidant assays were also examined to gain a more comprehensive understanding of antioxidant potential [36].

### 3.6. Use of Artificial Intelligence

Artificial intelligence (AI) tools were employed solely as supportive aids to enhance the interpretative clarity, linguistic precision, and overall presentation quality of the manuscript. All experimental procedures and data reported in this study were entirely produced by the authors through laboratory experimentation, without AI involvement in data generation. The AI’s contribution was strictly limited to improving textual coherence, readability, and technical accuracy. All AI-assisted elements were carefully reviewed, verified, and approved by the authors to ensure consistency with the study’s objectives and to uphold the highest standards of scientific rigor, transparency, and integrity.

## 4. Conclusions

This study comprehensively examined the chemical composition and biological effects of *E. laguroides* subsp. *laguroides*, demonstrating that different plant organs possess unique structural and functional characteristics. Stem samples showed the highest phenolic and flavonoid content, which was consistent with the strong antioxidant capacity observed in most tests. While leaves were similar to stems in total phenolic content, flowers were particularly notable for their various hydroxybenzoic and hydroxycinnamic acid derivatives. Roots, on the other hand, were characterized by a distinct phenolic profile, dominated by vanillin and chlorogenic acid-like compounds.

The strong agreement between antioxidant assays and their high correlation with total phenolic and flavonoid contents highlights the decisive role of polyphenols, particularly hesperidin and (+)-catechin, in the redox behavior of the extracts. In contrast, the weak correlation of metal ion chelating activity with other assays suggests a different mechanism for this effect.

The moderate enzyme inhibition observed in all parts of the plant indicates a balanced yet multifaceted biological activity. Although anti-cholinesterase and anti-tyrosinase effects are weaker than those of standard inhibitors, the consistent occurrence of these activities suggests that the plant may possess mild neuromodulatory and skin-protective potential. Furthermore, the strong positive correlation between tyrosinase inhibition and antioxidant activity supports the contribution of redox phenolics to this effect.

Overall, *E. laguroides* subsp. *laguroides* stands out as a phytochemically rich species with strong antioxidant capacity and moderate enzyme inhibitory effects. The chemical and biological differences observed between different organs indicate that careful consideration should be given to which organ to use in future pharmacological or nutritional supplement studies. Furthermore, purification of the predominant phenolic compounds, elucidation of their mechanisms of action, and further studies in living systems will provide a clearer picture of the biological potential of this endemic species.

## Figures and Tables

**Table 1:** Concentration (µg/g extract) of selected phenolic compounds in *E. laguroides* subsp. *laguroides* extracts.

No	Compounds	Flowers	Leaves	Stems	Roots
1	Hesperidin	11,819 ± 67 *^c^*	13,969 ± 27 *^b^*	18,016 ± 52 *^a^*	3788 ± 37 *^d^*
2	(+)-Catechin	1951 ± 59 *^b^*	1726 ± 19 *^c^*	4434 ± 10 *^a^*	1384 ± 1 *^d^*
3	(−)-Epicatechin	1882 ± 38 *^b^*	1010 ± 3 *^c^*	2238 ± 1 *^a^*	1895 ± 14 *^b^*
4	Protocatechuic acid	429 ± 8 *^a^*	84.0 ± 0.6 *^c^*	187 ± 2 *^b^*	64.5 ± 0.7 *^d^*
5	Hyperoside	426 ± 6 *^b^*	361 ± 1 *^c^*	497 ± 7 *^a^*	92.5 ± 2.9 *^d^*
6	Verbascoside	133 ± 2 *^c^*	69.1 ± 0.6 *^d^*	582 ± 25 *^a^*	184 ± 1 *^b^*
7	*p*-Coumaric acid	108 ± 2 *^a^*	12.4 ± 0.6 *^d^*	40.3 ± 1.0 *^c^*	47.5 ± 0.8 *^b^*
8	3-Hydroxybenzoic acid	100 ± 1 *^a^*	14.2 ± 1.1 *^d^*	64.3 ± 0.7 *^c^*	83.5 ± 0.7 *^b^*
9	4-Hydroxybenzoic acid	101 ± 1 *^a^*	14.0 ± 0.5 *^d^*	65.0 ± 1.7 *^c^*	81.4 ± 0.6 *^b^*
10	Ferulic acid	81.5 ± 1.1 *^a^*	19.2 ± 0.3 *^c^*	19.2 ± 0.1 *^c^*	33.9 ± 1.5 *^b^*
11	Syringic acid	57.8 ± 1.9 *^a^*	5.87 ± 0.07 *^d^*	25.2 ± 0.4 *^c^*	45.2 ± 0.3 *^b^*
12	Gallic acid	49.9 ± 0.7 *^a^*	32.3 ± 0.5 *^c^*	44.1 ± 0.1 *^b^*	14.3 ± 0.2 *^d^*
13	Quercetin	48.2 ± 0.7 *^a^*	16.2 ± 0.1 *^d^*	26.9 ± 0.3 *^b^*	21.9 ± 0.7 *^c^*
14	Rosmarinic acid	26.8 ± 0.7 *^a^*	17.8 ± 0.7 *^b^*	12.1 ± 0.1 *^d^*	14.5 ± 0.4 *^c^*
15	Caffeic acid	19.9 ± 0.3 *^a^*	2.91 ± 0.10 *^d^*	6.88 ± 0.40 *^c^*	9.9 ± 0.1 *^b^*
16	Vanillin	15.3 ± 0.4 *^c^*	5.25 ± 0.08 *^d^*	45.0 ± 0.9 *^b^*	61.0 ± 0.5 *^a^*
17	Taxifolin	14.9 ± 0.1 *^a^*	0.66 ± 0.08 *^d^*	2.39 ± 0.06 *^c^*	6.66 ± 0.21 *^b^*
18	Luteolin 7-glucoside	12.4 ± 0.4 *^b^*	5.24 ± 0.13 *^d^*	8.64 ± 0.13 *^c^*	15.7 ± 0.6 *^a^*
19	Chlorogenic acid	9.67 ± 0.21 *^c^*	8.22 ± 0.14 *^c^*	17.8 ± 0.1 *^b^*	103 ± 3 *^a^*
20	Sinapic acid	4.86 ± 0.07 *^b^*	1.52 ± 0.06 *^c^*	4.56 ± 0.09 *^b^*	13.1 ± 0.3 *^a^*
21	Eriodictyol	1.71 ± 0.01 *^c^*	0.41 ± 0.03 *^d^*	2.40 ± 0.02 *^b^*	3.69 ± 0.19 *^a^*
22	2-Hydroxycinnamic acid	nd	nd	nd	nd
23	3,4-Dihydroxyphenylacetic acid	nd	nd	nd	nd
24	Apigenin 7-glucoside	nd	nd	nd	nd
25	Apigenin	nd	nd	nd	nd
26	Kaempferol	nd	nd	nd	nd
27	Luteolin	nd	nd	nd	nd
28	Pinoresinol	nd	nd	nd	nd

Values are expressed as mean ± standard deviation (SD) of three independent measurements. Values indicated by the same superscripts (a–d) within the same row are not significantly different according to Tukey’s HSD test at the 5% significance level. Compounds listed as “nd” were included in the targeted LC–ESI–MS/MS analytical panel but were not detected above the limit of detection in the analyzed extracts.

**Table 2:** Antioxidant activities of *E. laguroides* subsp. *laguroides* extracts.

Assays	Flowers	Leaves	Stems	Roots	Trolox	EDTA
Phosphomolybdenum (EC_50_: mg/mL)	1.06 ± 0.003 ^cd^	1.06 ± 0.005 ^c^	0.85 ± 0.04 ^b^	1.19 ± 0.06 ^d^	0.46 ± 0.02 ^a^	-
CUPRAC reducing power (EC_50_: mg/mL)	0.73 ± 0.01 ^c^	0.68 ± 0.03 ^c^	0.51 ± 0.01 ^b^	1.55 ± 0.04 ^d^	0.17 ± 0.01 ^a^	-
FRAP reducing power (EC_50_: mg/mL)	0.30 ± 0.005 ^d^	0.27 ± 0.005 ^c^	0.19 ± 0.005 ^b^	0.60 ± 0.001 ^e^	0.049 ± 0.003 ^a^	-
DPPH radical (IC_50_: mg/mL)	1.52 ± 0.03 ^d^	1.24 ± 0.004 ^c^	0.94 ± 0.01 ^b^	3.29 ± 0.15 ^e^	0.27 ± 0.02 ^a^	-
ABTS radical cation (IC_50_: mg/mL)	0.95 ± 0.02 ^c^	0.85 ± 0.05 ^c^	0.58 ± 0.01 ^b^	1.49 ± 0.03 ^d^	0.17 ± 0.02 ^a^	-
Ferrous ion chelating (IC_50_: mg/mL)	5.74 ± 0.46 ^c^	na	3.84 ± 0.59 ^b^	6.26 ± 0.57 ^c^		0.020 ± 0.002 ^a^

EDTAE means ethylenediaminetetraacetic acid (disodium salt). Values indicated by the same superscripts (a–e) within the same row are not significantly different according to Tukey’s HSD test at the 5% significance level. na: not active.

**Table 3:** Enzyme inhibition activity of *E. laguroides* subsp. *laguroides* extracts.

Samples	AChE (IC_50_: mg/mL)	BChE (IC_50_: mg/mL)	Tyrosinase (IC_50_: mg/mL)	α-Amylase (IC_50_: mg/mL)	α-Glucosidase (IC_50_: mg/mL)
Flowers	1.06 ± 0.02 *^b^*	1.01 ± 0.02 *^b^*	1.08 ± 0.001 *^c^*	3.20 ± 0.06 *^b^*	1.00 ± 0.01 *^a^*
Leaves	1.30 ± 0.02 *^c^*	1.04 ± 0.01 *^b^*	1.08 ± 0.001 *^c^*	3.10 ± 0.06 *^b^*	0.98 ± 0.004 *^a^*
Stems	1.23 ± 0.07 *^c^*	1.04 ± 0.03 *^b^*	1.05 ± 0.003 *^b^*	3.10 ± 0.05 *^b^*	1.00 ± 0.003 *^a^*
Roots	1.02 ± 0.02 *^b^*	1.01 ± 0.02 *^b^*	1.13 ± 0.003 *^d^*	3.48 ± 0.01 *^c^*	1.02 ± 0.003 *^a^*
Galanthamine	0.0032 ± 0.0002 *^a^*	0.0031 ± 0.0003 *^a^*	-	-	-
Kojic acid	-	-	0.082 ± 0.002 *^a^*	-	-
Acarbose	-	-	-	0.95 ± 0.02 *^a^*	1.12 ± 0.030 *^b^*

Values indicated by the same superscripts (a–d) within the same column are not significantly different according to Tukey’s HSD test at the 5% significance level.

**Table 4:** Correlations among phenolic compounds and assays.

	TAP	DPPH	ABTS	CUPRAC	FRAP	FICA	AChEIA	BChEIA	TIA	AAIA	AGIA
DPPH	0.886										
ABTS	0.948	0.973									
CUPRAC	0.883	0.990	0.966								
FRAP	0.927	0.992	0.986	0.990							
FICA	0.526	0.181	0.370	0.251	0.291						
RACI	0.956	0.974	0.995	0.977	0.993	0.393					
AChEI	−0.570	−0.799	−0.703	−0.718	−0.732	0.364					
BChEIA	−0.564	−0.643	−0.565	−0.570	−0.588	0.216	0.793				
TIA	0.920	0.962	0.966	0.985	0.982	0.385	−0.612	−0.491			
AAIA	0.642	0.909	0.812	0.916	0.864	−0.081	−0.767	−0.593	0.855		
AGIA	0.396	0.734	0.589	0.724	0.667	−0.395	−0.753	−0.507	0.650	0.906	
Total flavonoid	0.736	0.951	0.881	0.910	0.906	−0.107	−0.934	−0.718	0.835	0.920	0.820
Total phenolic	0.922	0.989	0.982	0.992	0.999	0.298	−0.716	−0.557	0.988	0.867	0.673
Hesperidin	0.850	0.993	0.951	0.993	0.984	0.148	−0.778	−0.594	0.966	0.935	0.784
(+)-Catechin	0.957	0.819	0.919	0.825	0.869	0.668	−0.448	−0.439	0.866	0.555	0.232
(−)-Epicatechin	0.463	0.066	0.257	0.130	0.183	0.980	0.438	0.218	0.273	−0.219	−0.510
Protocatechuic acid	0.200	0.169	0.148	0.285	0.232	0.363	0.296	0.267	0.385	0.247	0.250
Hyperoside	0.801	0.913	0.868	0.950	0.925	0.239	−0.579	−0.440	0.960	0.889	0.764
Verbascoside	0.843	0.598	0.754	0.602	0.666	0.797	−0.231	−0.283	0.664	0.276	−0.080

Data show the Pearson Correlation Coefficients between the parameters. TAP: total antioxidant activity by phosphomolybdenum method. AAIA, AGIA, AChEIA, BChEIA and TIA: α-amylase, α-glucosidase, acetylcholinesterase, butyrylcholinesterase and tyrosinase inhibition activities, respectively. ABTS and DPPH: ABTS and DPPH radical scavenging activities, respectively. CUPRAC and FRAP: CUPRAC and FRAP reducing power potential, respectively. FICA: Ferrous ion chelating activity. RACI: Relative antioxidant capacity index.

## Data Availability

All data generated or analyzed during this study are included in this published article.

## Supplementary Materials

The following supporting information can be downloaded at: https://www.mdpi.com/article/10.3390/molecules31050826/s1, Figure S1: Representative LC–ESI–MS/MS chromatograms of methanolic extracts obtained from different organs of *E. laguroides* subsp. *laguroides*: (A) flower, (B) leaf, (C) stem, and (D) root. The numbered peaks correspond to the following identified phenolic compounds: (1) protocatechuic acid, (2) (+)-catechin, (3) (−)-epicatechin, and (4) hesperidin.; Table S1: ESI–MS/MS Parameters and analytical characteristics for the Analysis of Target Analytes by MRM Negative and Positive Ionization Mode; Table S2: Calibration curves and sensitivity properties of the method.
